# High spatial and temporal variability in Antarctic ice discharge linked to ice shelf buttressing and bed geometry

**DOI:** 10.1038/s41598-022-13517-2

**Published:** 2022-06-29

**Authors:** Bertie W. J. Miles, Chris R. Stokes, Stewart S. R. Jamieson, Jim R. Jordan, G. Hilmar Gudmundsson, Adrian Jenkins

**Affiliations:** 1 School of Geosciences, Edinburgh University, Edinburgh, EH8 9XP UK; 2 Department of Geography, Durham University, Durham, DH1 3LE UK; 3 Department of Geography and Environmental Sciences, Northumbria University, Newcastle upon Tyne, NE1 8ST UK; 4 Laboratoire de Glaciologie, Université Libre de Bruxelles, Brussels, Belgium

**Keywords:** Climate sciences, Cryospheric science

## Abstract

Antarctica’s contribution to global mean sea level rise has been driven by an increase in ice discharge into the oceans. The rate of change and the mechanisms that drive variability in ice discharge are therefore important to consider in the context of projected future warming. Here, we report observations of both decadal trends and inter-annual variability in ice discharge across the Antarctic Ice Sheet at a variety of spatial scales that range from large drainage basins to individual outlet glacier catchments. Overall, we find a 37 ± 11 Gt year^−1^ increase in discharge between 1999 and 2010, but a much smaller increase of 4 ± 8 Gt year^−1^ between 2010 and 2018. Furthermore, comparisons reveal that neighbouring outlet glaciers can behave synchronously, but others show opposing trends, despite their close proximity. We link this spatial and temporal variability to changes in ice shelf buttressing and the modulating effect of local glacier geometry.

## Introduction

Since 1992, the Antarctic Ice Sheet (AIS) has contributed 7.6 mm to global mean sea level rise and this contribution is accelerating^1^. This imbalance has been caused by increased ice discharge^2–5^, particularly in the West Antarctic Ice Sheet (WAIS), outweighing longer-term trends of increased precipitation, particularly over the East Antarctic Ice Sheet (EAIS)^1,6^. Thus, in the context of predicted future warming^7^, it is important to understand the spatial and temporal variability in ice discharge from Antarctica and the likely drivers of recent and future dynamic changes, which remain a key uncertainty in future projections of sea level rise^8^.

Several estimates of the change in ice discharge from Antarctica have shown that the increase over recent decades has been dominated by the WAIS, where ice discharge has increased by 30 ± 8 Gt year^−1^ between ~ 2008 and 2015^5^, or by 48 Gt year^−1^ between 1999 and 2010 and 2010–2017^3^. In the East Antarctic Ice Sheet (EAIS) there has been comparatively little change, with estimates suggesting − 1 ± 11 Gt year^−1^ decrease^5^ in ice discharge between ~ 2008 and 2015, and a 4 Gt year^−1^ increase in ice discharge between 1999–2010 and 2017^3^. However, these studies mostly focus on decadal-scale changes in ice discharge from large regional drainage basins that sometimes average the contribution from several major outlet glaciers, thereby potentially masking both spatial and temporal variability. In contrast, few studies have focussed on the inter-annual variability in ice discharge within these basins or investigated changes at the scale of individual outlet glacier catchments. Establishing the magnitude of any inter-annual or spatial variability is important because it may enable an improved understanding of longer-term trends in ice discharge and the processes that are driving such variability. This is also relevant to numerical modelling studies that use modern observations to help constrain Antarctica’s future contribution to mean global sea level rise^9^.

In this study, we compile annual ice velocity products^10,11^ to create 12 time-stamped ice velocity mosaics spanning from 1999–2006 to 2018 to approximate ice discharge over both decadal and inter-annual time periods. We report changes in ice discharge at both previously determined^12^ regional scale drainage basins and individual glacier catchments. We show that the rate of change in ice discharge from Antarctica throughout our observational period has fluctuated through time, but also highlight considerable spatial and temporal variability where neighbouring catchments can simultaneously undergo opposing trends. We then explore the drivers of this variability, specifically analysing the role of changes in ice shelf buttressing and how feedbacks between ice discharge and geometry modulate the overall response of individual glaciers.

## Methods

### Mosaic generation

We use the annual MEaSUREs^10^ and ITS_Live^11^ velocity mosaics of Antarctica to create time-stamped composite velocity mosaics. The ITS_Live mosaics are available annually between 1999 and 2018 and have a 240 m spatial resolution. The mosaics are weighted averages of all Landsat-7 and Landsat-8 velocity image pairs with a centre date in each calendar year^11^. The MEaSUREs mosaics are available from 2005–2006 to 2016–2017 and have a 1 km spatial resolution. They are weighted averages of all velocity image pairs from a variety of sensors (ALOS PALSAR, RADARSAT, ENVISAT, Terra-SAR-X, Sentinel-1 and Landsat-8) with a centre date between July and June^10^. We first resampled the ITS_Live mosaics to a spatial resolution of 1 km to match the MEaSUREs velocity mosaics. To maximise spatial coverage, we then group the annual mosaics (ITS_Live and MEaSUREs) into epochs, before creating composite mosaics of both datasets by taking the average pixel value in all mosaics in each epoch. Prior to 2013, mosaics are grouped as follows: 1999–2002, 2003–2006, 1999–2006, 2007–2008, 2009–2010 and 2011–2012 (Table S1; Fig. S1), from 2013 mosaics are at an annual resolution (Table S1; Fig. S1) because of the greater spatial coverage provided by the launch of the Landsat-8 satellite.

### Ice discharge

We produce a time series of ice discharge from 1999–2006 to 2018 for the EAIS and WAIS, for each of the 15 commonly used regional scale basins^1^, and then for 148 major outlet glacier catchments^3^. Due to the 1 km spatial resolution of each velocity mosaic and the poor performance of the velocity products in the narrow fjords of the Antarctic Peninsula^5^, we focus our analysis on the EAIS and WAIS. We first calculate a reference ice discharge dataset using the MEaSUREs v2 ice velocity mosaic of Antarctica^13^ which has complete spatial coverage, but is not time-stamped. We calculate ice discharge along an amended version of GL0 discharge gate used in Ref.^5^, which is located at or slightly behind the grounding line, and which remains fixed throughout our study. We amended the discharge gate by moving it slightly inland in locations with small or no ice shelves that had retreated during the observational period (e.g. Frost and Cook West glaciers) meaning the discharge gate was previously located over the ocean.

For ice thickness, we use the BedMachine v1 dataset^14^. We do not correct for the vertical velocity gradient of ice at the grounding line or any dynamic changes in ice thickness that might have taken place over the study period. We justify this approach because our study is largely focussed on inter-annual variations in velocity that predominantly drive changes in ice discharge, rather than absolute changes in mass. As in most previous studies^3,4^, we do not correct for either ice surface elevation change or grounding line migration. Pan-ice sheet surface elevation time series are available^15^, but for most glacier catchments changes in surface elevation over our observational time period (1999–2018) make little difference to ice discharge (Fig. S2). Furthermore, any changes in surface elevation at the grounding line are likely to result in grounding line migration, which in turn alters ice thickness as bedrock depth changes. In the absence of any suitable grounding line products, we decide to assume a constant ice thickness. Ice discharge, *F*, is calculated at each node in the discharge gate according to:

1 $$F=\left[ {V}_{s}W T sin\theta D\right]$$

*W* is the width of the flux gate, *T* is the ice thickness, $\theta$ is the angle between the velocity vector and the angle of the flux gate, *D* is the assumed density of ice, 917 kg m^−3^ and *V*_*s*_ the ice speed calculated by:

2 $${V}_{s}=\sqrt{{{v}_{x}}^{2}+{{v}_{y}}^{2}}$$

where *v*_*x*_ and *v*_*y*_ are the x and y components of velocity respectively. We then sum the ice discharge in each node within each catchment, basin and ice sheet which are defined by the MEaSUREs Antarctic boundaries^12^. To calculate the ice discharge time-series we use the same equation, changing only the values dependent on velocity, *v*_*x*_*, v*_*y*_ and $\theta$, which are extracted from each of the time-stamped mosaics. For nodes where no velocity data is available, we revert to the corresponding node in the reference dataset, meaning that in some cases a given catchment may have a mixture of time-stamped and reference data. The percentage of ice discharge derived from the reference velocity product and time-stamped data for each individual catchment over each epoch is presented in the supplementary dataset. For some slower flowing outlet glaciers, their discharge is sensitive to small erroneous directional changes in velocity at the shear margins^5^. To account for this we used the reference discharge data when $sin\theta$ was less than 0.3. The final dataset detailing the ice discharge for each catchment, basin and ice sheet throughout our epochs is available in the supplementary dataset.

### Uncertainties

Sources of uncertainty in our ice discharge time-series emanate from uncertainties in the velocities and ice thickness at the discharge gate. We the use the accompanying uncertainty grids of the annual ITS_Live and MEaSUREs velocity mosaics to form the basis of our velocity uncertainty estimates. These uncertainty grids are statistical estimates based on the image sensor type, image pair count, and time separation between image pairs. Further details on the processes used to estimate these uncertainties can be found in^10^ and^11^. We then merge these uncertainty mosaics to create a composite of the average uncertainty for each epoch used in the same manner as the velocity mosaics described above. We calculate the uncertainty in velocity at each node by using a range of values in the x and y components of the velocity, *v*_*xr*_ and *v*_*yr*_, defined as:

3 $${v}_{xr}=\left[\left({v}_{x}-{v}_{xerr}\right)\le x\le \left({v}_{x}+{v}_{xerr}\right)\right]$$

4 $${v}_{yr}=\left[\left({v}_{y}-{v}_{yerr}\right)\le y\le \left({v}_{y}+{v}_{yerr}\right)\right]$$

where *v*_*x*_ and *v*_*y*_ are the x and y velocity values and *v*_*yerr*_ and *v*_*xerr*_ are the uncertainty values. We then use a random number generator using a uniform distribution to choose an integer in each of the ranges of *v*_*xr*_ and *v*_*yr*_, which are then fed into Eq. (1) as the velocity values *v*_*x*_ and *v*_*y*_. If there is no time-stamped data available at a given epoch we revert to both the reference velocity and uncertainty mosaics. We then repeat this process 100 times, with the final uncertainty at each node taken as the difference between the maximum and minimum ice discharge from the 100 simulations. This method of estimating uncertainty simultaneously incorporates any uncertainty associated with ice speed, but also any uncertainty associated with the velocity vector in relation to the angle of the flux gate, which may be particularly important over slower flowing regions. To then estimate the error for each catchment, basin and ice sheet, we propagate velocity uncertainties, which is the square root of the sum of the velocity uncertainties of each node squared. Finally, we add an arbitrary 1% of the reference discharge to uncertainties at each catchment to account for combining the ITS_Live dataset and MEaSUREs datasets, which are derived from different sensors. The velocity uncertainty for each catchment, basin and ice sheet are detailed in the supplementary dataset. Importantly, these velocity uncertainties should be treated as qualitative^11^ and the confidence of any given data point should not be based on the quantitative estimates of error alone. To judge the confidence in any changes in ice discharge, both the consistency of the time series in ice discharge and the spatial pattern of velocity change should also be considered in addition to our uncertainty estimates.

We use the accompanying uncertainty grid in the BedMachine dataset to form the basis of our ice thickness uncertainties. To calculate the uncertainties we use Eq. (1), but replace $T$, ice thickness, with *T*_*err*_, ice thickness uncertainty. We then calculate the ice thickness uncertainty for each catchment by propagating the uncertainty at each node, in the same manner as the velocity uncertainties. The error associated with ice shelf thickness for each catchment, basin and ice sheet are shown in the supplementary dataset. The combined uncertainty for velocity and ice shelf thickness is detailed as follows:

5 $${F}_{err}=\sqrt{{{F}_{verr}}^{2}+{{F}_{terr}}^{2}}$$

where *F*_*err*_ is the total uncertainty, *F*_*verr*_ is the uncertainty associated with velocity and *F*_*terr*_ is the uncertainty associated with ice thickness. The combined errors are detailed in the supplementary dataset.

### Ice discharge timeseries

We calculate ice discharge time-series at ice sheet, basin and catchment scales. At the ice sheet and basin scale, the time-series incorporates both reference data and time-stamped data. At a catchment scale we produce a filtered time-series. This time-series removes any data points where the spatial coverage of time-stamped data is less than 70%. This is done to make sure that any variations in ice discharge reflect time-stamped data as opposed to the reference data. In this time-series, we also remove any erroneous data points, which are classified as those data-points that fall outside of 1.5 standard deviations of the residuals of trendline for each catchment, calculated using a cubic spline. This timeseries is used to create Fig. 4 and the removal of erroneous data points explains why there are some gaps in the timeseries. We note that a total of 321 data points (18% of all data points) are classified as erroneous outliers using our 1.5 standard deviation criterion. The majority of these data points (185 or 58%) are concentrated on very small catchments with a mean discharge of less than 3 Gt year^−1^. The reason most outliers are concentrated in these catchments is because ice typically flows at slower speeds here and results in relatively higher uncertainties. Outlet glaciers in these catchments are also typically smaller, meaning the 1 km spatial resolution of the velocity data may not be sufficient to accurately capture any velocity variations. A further 53 (16%) of these outliers are located on the glaciers that feed the Ross and Filchner-Ronne ice shelves. These glaciers are located closer to the South Pole meaning there is a much poorer image coverage, particularly earlier on in the time series, which also increases uncertainty. The remaining 83 outliers (26%) are typically located on other slower flowing regions e.g. Dronning Maud Land.

### Ice shelf thickness and ice discharge anomalies

We use the ice shelf thickness time-series from^16^ to extract time-series in ice shelf thickness change. This dataset has a spatial resolution of 10 km and has been corrected for changes in firn content. It spans from 1992 to 2018 and has a data point every 3 months. We extract the average ice shelf thickness change from all floating ice in our example catchments. The only exception to this is at Denman Glacier, where we only extract thickness change from the fast flowing Denman trunk and not the neighbouring Shackleton Ice Shelf. We then linearly de-trend the resulting time-series in ice shelf thickness change, before fitting a cubic spline (Fig. S3). We then linearly de-trend our time-series in ice discharge and then compare the ice shelf thickness anomalies to ice discharge anomalies.

## Results

### Spatial and temporal variability in ice discharge

**Figure 1:**
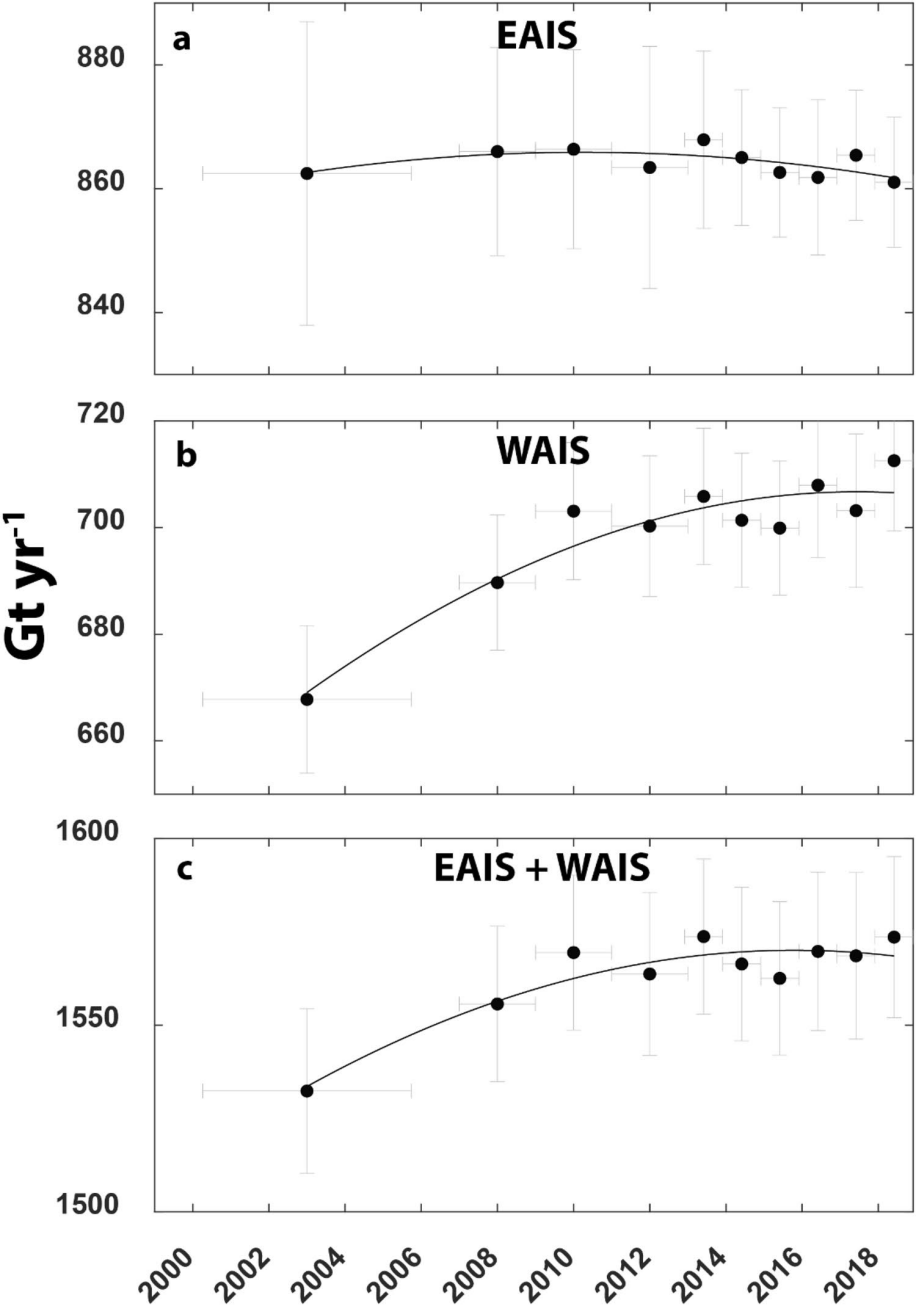
Time series of ice discharge from (**a**) East Antarctic Ice Sheet (EAIS). (**b**) West Antarctic Ice Sheet (WAIS) and (**c**) Combined EAIS and WAIS. All fitted with a Gaussian best fit model in black.

Between 1999–2006 and 2018 we find a small − 4 ± 7 Gt year^−1^ decrease in the EAIS (Fig. 1a) and a 45 ± 8 Gt year^−1^ increase in the annual ice discharge of the WAIS (Fig. 1b). Notably, the rate of acceleration in ice discharge of the WAIS has not been consistent through time. Annual ice discharge increased by 35 ± 8 Gt year^−1^ between 1999–2006 and 2009–2010. Since ca. 2010, however, there has been a much more limited increase of 9 ± 6 Gt year^−1^ between 2009–2010 and 2018. In the EAIS we observe considerably less temporal variability and throughout all epochs, ice discharge does not deviate by more than ± 4 Gt yr^-1^ from the overall mean (Fig. 1a). Taken together, our results are in agreement with previous work that shows that the increase in ice discharge from Antarctica has been dominated by the WAIS, with a negligible contribution from the EAIS^3,5^. However, our results also highlight that since 2010, their combined annual ice discharge has only increased by a modest 4 ± 8 Gt year^−1^ (Fig. 1c).

**Figure 2:**
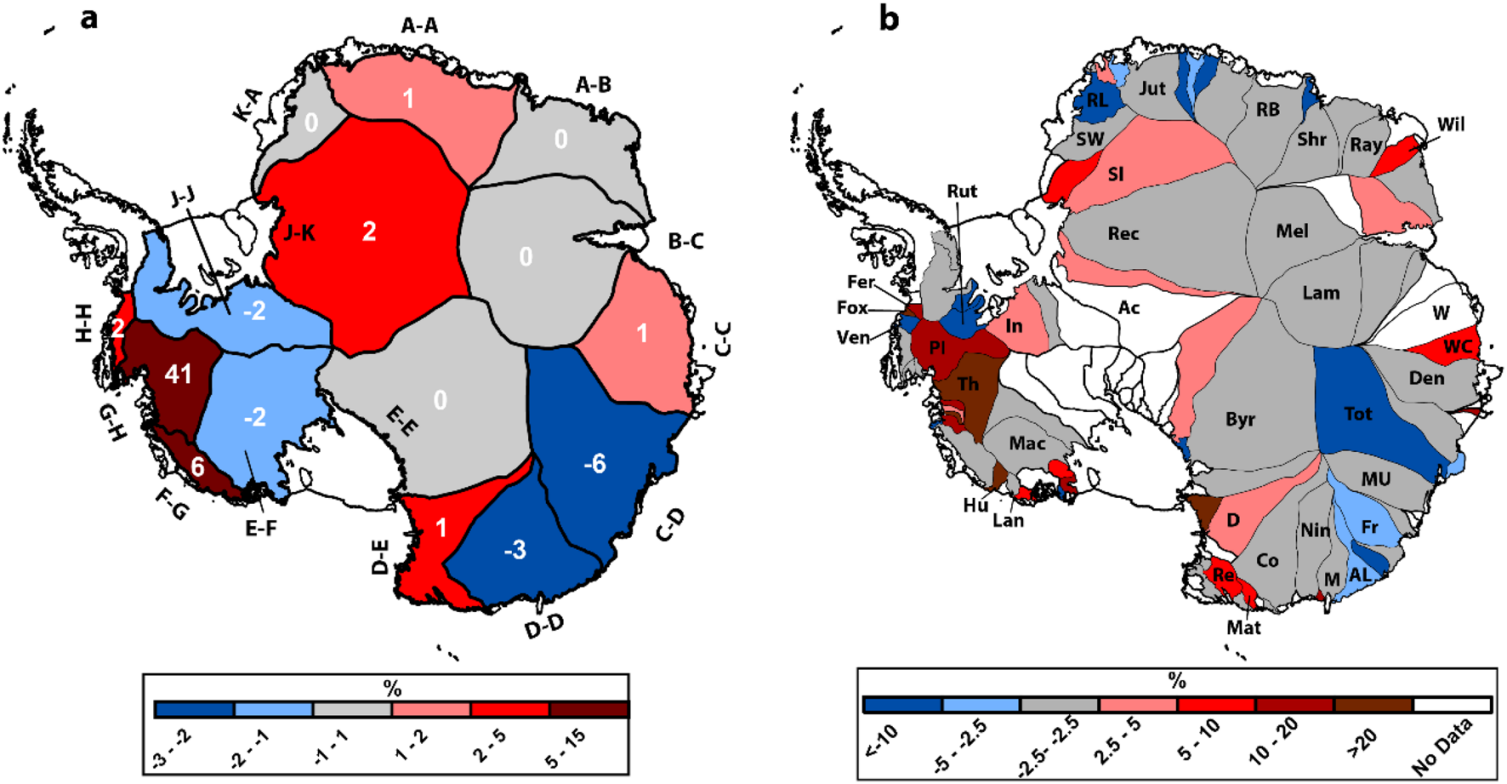
(**a**) Percentage change in ice discharge between 1999–2005 and 2018 for each IMBIE drainge basin^12^, the white numbers are the change in Gt year^−1^. (**b**) Percentage change in ice discharge between 1999–2005 and 2018 for each individual catchment^3^: Ferrigno (Fer), Fox (Fox), Vennable (Ven), Pine Island (PI), Thwaites (Th), Hull (Hu), Land (Lan), MacAyeal (Mac), Byrd (Byr), David (D), Rennick (Re), Matusevich (Mat), Cook (Co), Ninnis (Nin), Mertz (M), Adelie Land (AL), Frost (Fr), Moscow University (MU), Totten (Tot), Denman (Den), Wilhelm II Coast (WC) West (W), Lambert (Lam), Mellor (Mel), Wilma (Wil) Rayner (Ray), Shr (Shirase), Roi Baudouin (RB), Jutulstraumen (Jut), Riiser-Larsen (RL), Stancomb-Wills (SW), Slessor (Sl), Reocvery (Rec), Academy (Ac), Insititue (In).

When examining the 15 regional drainage basins (Fig. 2a), it is clear that the dominant signal of increased ice discharge emanates from the Amundsen Sea sector of the WAIS, where we report a 15 ± 1% (41 ± 4 Gt year^−1^) increase in ice discharge in basin G–H between 1999–2006 and 2018 (Fig. 2a). Elsewhere in the WAIS, we observe a 7 ± 3% (6 ± 3 Gt year^−1^) increase in Marie Byrd Land (basin F–G), a smaller 4 ± 2% (2 ± 1 Gt year^−1^) increase in basin H–H (Fig. 2a), and a decrease of − 2 ± 3% (− 2 ± 3 Gt year^−1^) in basin E–F, which feeds the Ross Ice Shelf. This is in addition to a − 2 ± 4% (− 2 ± 6 Gt year^−1^) decrease in discharge at basin J–J, which feeds the Ronne Ice Shelf. In the EAIS, we observe increases in ice discharge in basin D–E (3 ± 2%; 1 ± 1 Gt year^−1^) and J–K (2 ± 2%; 2 ± 2 Gt year^−1^) (Fig. 2a). These increases are balanced by decreases in Wilkes Land of − 3 ± 2% (− 6 ± 3 Gt year^−1^) in basin C–D and − 3 ± 2% (− 3 ± 2 Gt year^−1^) in basin D-D (Fig. 2a) between 1999–2006 and 2018 (Fig. 2a).

**Figure 3:**
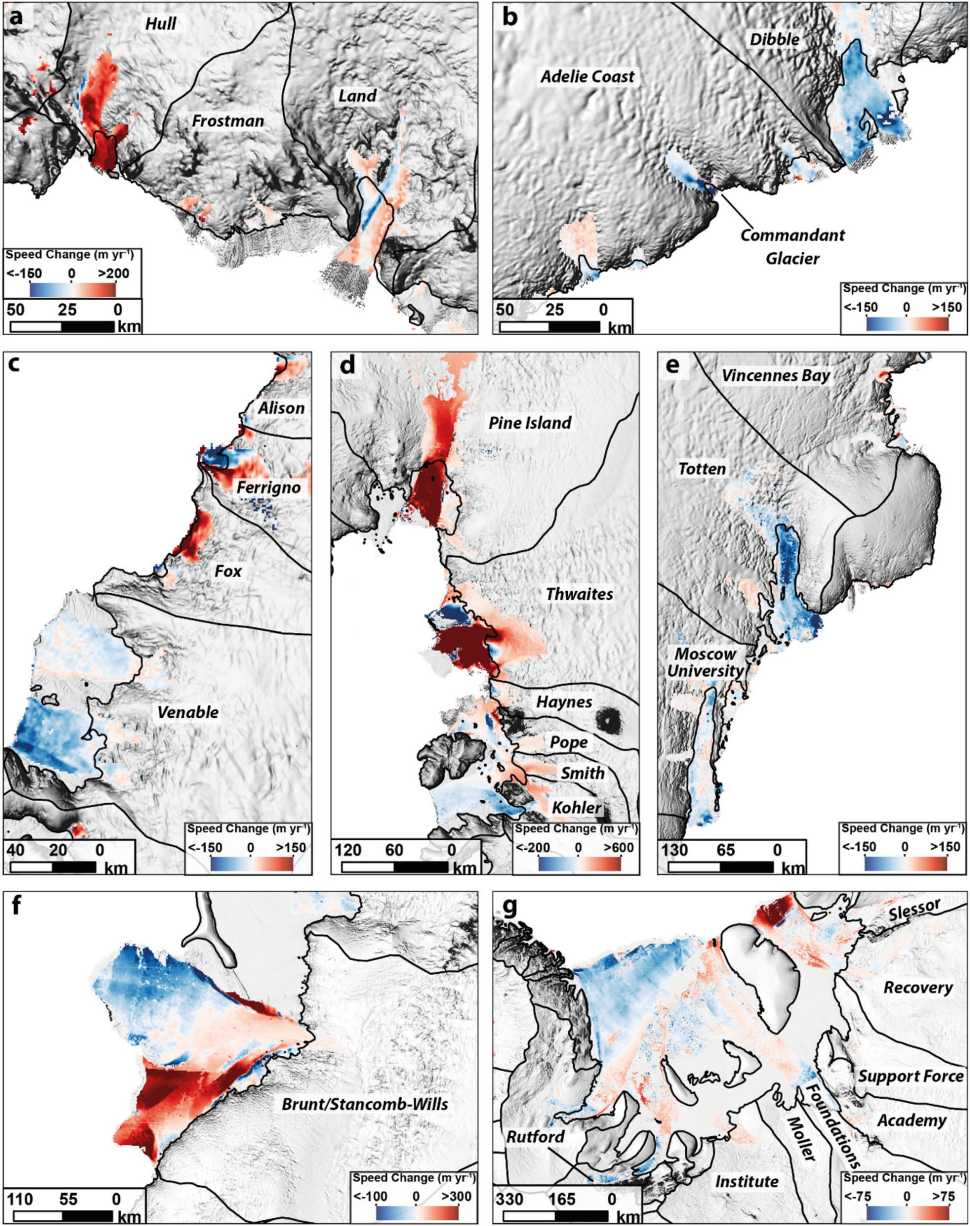
Spatial pattern of ice speed change between 1999–2006 and 2018 where ice flow speed is greater than 200 m year^−1^ for (**a**) Land and Hull catchments (**b**) Adelie Coast and Dibble catchments (**c**) Venable, Fox and Ferrigno catchments (**d**) the Amundsen Sea sector, (**e**) Totten and Moscow University catchments (**f**) Brunt/Stancomb-Wills catchment and (**g**) The Filchner-Ronne Ice Shelf. All velocity maps are overlain on the REMA mosaic^61^. Figure was generated using ArcMap GIS software (https://www.arcgis.com).

We observe a more nuanced pattern of spatial changes when ice discharges are measured from individual outlet catchments (Fig. 2b). This reveals that changes in ice discharge in individual catchments do not necessarily follow the wider regional scale patterns and neighbouring catchments can simultaneously undergo opposing patterns (Fig. 2b). For example, in basin H–H in the Bellingshausen Sea, ice discharge in the Venable catchment has decreased by − 10 ± 3%, but ice discharge has increased by 95 ± 20% in the neighbouring Fox catchment (Figs. 2b, 3c). We also observe complex patterns of change in basin G-H in the Amundsen Sea, where we observe large accelerations in discharge from the Pine Island (14 ± 2%), Thwaites (21 ± 2%), Haynes (14 ± 5%), Smith (29 ± 6%) and Kohler (12 ± 6%) catchments, but a much more limited increase in the Pope catchment (4 ± 6%) and clear slow-downs across parts of the ice shelves in this large catchment (Figs. 2b, 3d). In Marie Byrd Land, basin F–G, we observe a 27 ± 6% increase in the Hull Glacier catchment, but little change in the neighbouring Land catchment (2 ± 4%) (Figs. 2b, 3a). We also observe very little change in most of the outlet glaciers feeding the Ross and Ronne ice shelves, apart from the decreases in ice discharge from the Whillans (− 13 ± 12%) and Rutford (− 7 ± 4%) catchments (Figs. 2b, 3d).

In the EAIS, and despite an overall increase in ice discharge from Basin D–E, most catchments remain unchanged throughout the observational period, with the bulk of the increase from D–E emanating from the David (5 ± 6%), Rennick (10 ± 7%) and Matusevich (7 ± 7%) glaciers (Fig. 2b). In basin D–D, we find little change in the ice discharge across the three largest catchments: Cook (− 1 ± 2%), Ninnis (0 ± 3%) and Mertz (− 1 ± 5%). Rather, the reduction in discharge from basin D–D has been driven by decreases at the much smaller Dibble (− 6 ± 3%) and Adelie Coast (− 4 ± 3%) catchments (Figs. 2b, 3b). In basin C-D, Wilkes Land, the decrease in ice discharge can be largely attributed to the largest outlet glacier (Totten), where ice discharge decreased by − 8 ± 2% (Figs. 2b, 3e). Elsewhere in the EAIS, we observe very little change within the catchments of basins B–C, A–B, A–A and K–A (Fig. 3f). The only exception to this is the Wilma-Robert-Downer catchment, where ice discharge has increased by 6 ± 5% (Fig. 2b). The increase in ice discharge at basin J-K, which feeds the Filchner Ice Shelf, can be explained by increases at the Slessor (4 ± 4%) and Institute (4 ± 6%) catchments (Figs. 2b, 3g).

**Figure 4:**
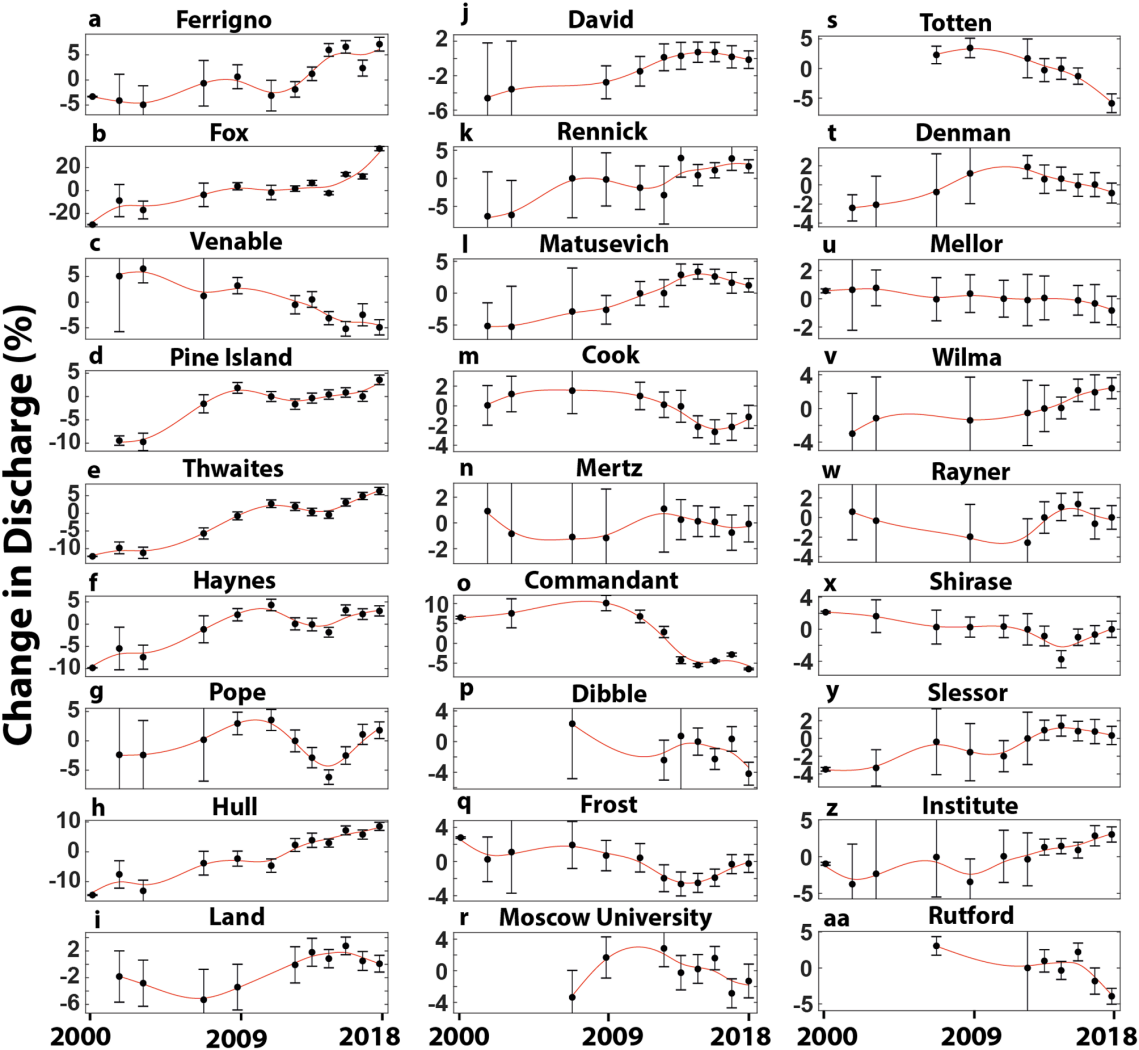
Time series of percentage change in ice discharge rates relative to the mean for selected catchments in West and East Antarctica (note different scales on the *y*-axes). Data for all catchments is available in the Supplementary dataset.

In addition to the marked spatial variability, we also observe temporal variations in ice discharge across individual catchments (Fig. 4). In the WAIS, for example, we find marked contrast in patterns of change between the neighbouring Ferrigno, Fox and Venable catchments (Fig. 4a–c). Ice discharge from the Fox catchment (Fig. 4b) increased at a near linear rate, which is in contrast to the Venable catchment (Fig. 4c), where ice discharge decreased at a near linear rate over the same period. At the Ferrigno catchment (Fig. 4a) we observe limited change between 2000 and 2010, before a 9 ± 2% increase in ice discharge between 2011–2012 and 2015. In the Amundsen Sea sector we report very similar patterns in ice discharge variation at Pine Island, Thwaites, Haynes and Smith catchments (Fig. 4d–g), where ice discharge rapidly increased between 2006 and 2012, before decreasing between 2012 and 2016. In Marie Byrd Land, we report a near constant acceleration of the Hull catchment (Fig. 4h), but a much more varied behaviour at the neighbouring Land catchment (Fig. 4i), where ice discharge decreased by − 4 ± 5% between 1999–2006 and 2007–2008 before increasing 9 ± 4% between 2007–2008 and 2016.

We also observe substantial temporal variability across individual catchments in the EAIS. At the Rennick (Fig. 4k) and Matusevich (Fig. 4l) catchments, we observe periods of both rapid acceleration and more gradual slowdowns in ice discharge. In the Cook catchment, ice discharge decreased by − 4 ± 2% between 2007/2008 and 2016, before increasing by 2 ± 2% between 2016 and 2018 (Fig. 4m). Ice discharge at the Commandant Glacier, which is located within the Adelie Land catchment accelerated by 3 ± 8% between 1999–2002 and 2009–2010 before undergoing a marked − 15 ± 2% decrease in ice discharge between 2009–2010 and 2018 (Fig. 4o). No other outlet glaciers within the Adelie Land catchment display similar variations in ice discharge despite their close proximity, further emphasising the considerable spatial and temporal variability. At the Frost catchment, ice discharge decreased by − 4 ± 2% between 2007/2008 and 2014, before increasing by 3 ± 2% between 2014 and 2018 (Fig. 4q). Ice discharge at the Moscow University (Fig. 4r), Totten (Fig. 4s) and Denman (Fig. 4t) catchments displayed near synchronous patterns, with ice discharge rates peaking around 2010, before decreasing between 2009/2010 and 2018. At the Rayner catchment, ice discharge gradually decreased by − 3 ± 7% between 2003/2006 and 2013, before rapidly increasing by 4 ± 2% between 2013 and 2016 (Fig. 4w). The overall increase in ice discharge at the Slessor catchment can be explained by a 5 ± 4% increase between 1999–2006 and 2015 (Fig. 4y). Overall, across the entire AIS, the contrasting spatial and temporal variability in ice discharge rates suggests that differing localised and regional scale drivers may be important in determining these patterns.

## Discussion

### Ice discharge change in Antarctica

Several studies have recently reported ice discharge change across Antarctica over a variety of different timescales, using slightly different methodologies and velocity products. In this section, we briefly compare our results to previously published estimates of ice discharge change and explore the potential reasons behind any differences. In the WAIS we observe a 45 ± 8 Gt year^−1^ increase in ice discharge between 1999–2006 and 2018. Over a comparable time period, a recent study^3^ observed a larger (66 Gt year^−1^) increase in ice discharge. Much of this discrepancy can be explained by the Pope, Smith and Kohler catchments (referred to as the Dotson and Crosson Ice Shelves in Ref.^3^) where Ref.^3^ collectively observed a 51% (21 Gt year^−1^) increase in discharge, while we report a much smaller 15% (6 Gt year^−1^) increase. The reason for this difference could be a consequence of the complex velocity patterns over the Crosson and Dotson ice shelves where sections of floating and grounded ice have been both accelerating and decelerating (Fig. 3d). Ice discharge change in Ref.^3^ was calculated using a scaling factor over the fastest flowing sections of the ice to estimate changes in discharge, meaning the full spatial variability in ice flow across the catchment may not be fully captured.

A separate study^5^ reported a 30 ± 8 Gt year^−1^ increase in ice discharge in the WAIS between the MEaSUREs (~ 2008) velocity mosaic and a velocity mosaic based on image pairs from 2013 to 2016. We report a smaller increase of 14 Gt ± 8 Gt year^−1^ between 2007–2008 and 2013–2016. The largest differences in discharge change between our results and Ref.^5^ come from basin J–J that feeds the Ronne Ice Shelf and basin F–G in Marie Byrd Land. In basin J–J we observe a decrease in discharge of − 3 Gt year^−1^, while Ref.^5^ observed a 2 Gt year^−1^ increase. This small difference could be related to the underlying data in the MEaSUREs velocity mosaic used in Ref.^5^. While most of the velocity measurements are from around 2008, velocities from the late 1990s and 2009 are used for glaciers that feed the Ronne Ice Shelf^13^. At basin F-G we observe a 6 Gt year^−1^ increase, while Ref.^5^ observed a 12 Gt year^−1^ increase in discharge. The reasons for this discrepancy remain unclear but we note that, over the same timespan (2008 to 2013–2016), Ref.^3^ reported only a 5 Gt year^−1^ increase in ice discharge at basin F-G, which is more consistent with our estimate. In the EAIS, our observations of limited change throughout the study period is consistent with the majority of previous studies^3,5^. The only exception to this is Ref.^17^ who reported a much larger increase in ice discharge in the EAIS (upwards of 50 Gt year^−1^) for the period 2008 to 2015. The underlying reasons as to why the ice discharge change reported by Ref.^17^ differs markedly to our results and other studies^3,5^ remains unclear.

The majority of the regional trends we observe in ice discharge between 1999–2006 and 2018 are consistent with reported mass balance trends i.e. mass loss in WAIS and limited change in the EAIS^1,3,5,6^. This further reinforces the notion that changes in ice discharge have been driving the observed changes in the mass balance of WAIS, as oppose to surface mass balance, at least over decadal time periods^1,3,5,6^. In the EAIS our observation of limited change in ice discharge is consistent with reconciled estimates of a mass balance that is close to zero, albeit with large uncertainties^1^. Indeed, there is a considerable range in mass balance estimates of the EAIS, typically depending on the methods used, with some studies estimating a positive mass balance^5,6^ and others a more negative mass balance^3^. The only regional exception where trends in mass balance do not appear to be consistent with trends in ice discharge is in Wilkes Land where we observe a decrease in ice discharge between 1999–2006 and 2018. However, multiple studies have confirmed that this region has continued to lose mass over roughly the same time period^1,3,5,6,18^, suggesting that the decrease in ice discharge in Wilkes Land has not been enough to reverse the trend of mass loss. The decrease is in ice discharge that we detect is focused at the Totten catchment and much of the decrease in ice discharge has occurred in recent years (2015–2018; Fig. 4s). We note that a reduction in ice discharge of a similar magnitude (around 11%) has been reported at Totten between 1996 and 2000^19^, likely caused by intermittent contact between its ice shelf and bed obstacles^20^. This demonstrates that our observed reduction in discharge is not unprecedented over longer multi-decadal timescales and further highlights the considerable interannual variability in some of the outlet glaciers in Wilkes Land.

### Drivers of ice discharge variability

Observations and numerical modelling experiments have shown that changes in ice discharge from marine-terminating outlet glaciers are predominantly controlled by changes in ice shelf buttressing, which can manifest in response to changes in ice shelf thickness^21,22^, ice shelf extent^23^ and the structural integrity or ‘damage’ of the ice shelf ^24^. Following a perturbation in buttressing, ice discharge responds instantaneously^21,22^, and the thickness changes induced, combined with the glacier’s unique geometrical setting, then determine the overall magnitude of the ensuing change in ice discharge^25–28^. These associated feedbacks are transient and the response time for a glacier to reach a theoretical steady-state following a perturbation in ice shelf buttressing is dependent on its unique geometry^26^. In the following sections we highlight some examples as to how these constantly evolving processes are controlling the inter-annual and spatial variability in ice discharge that we have observed.

#### Ice shelf thickness

**Figure 5:**
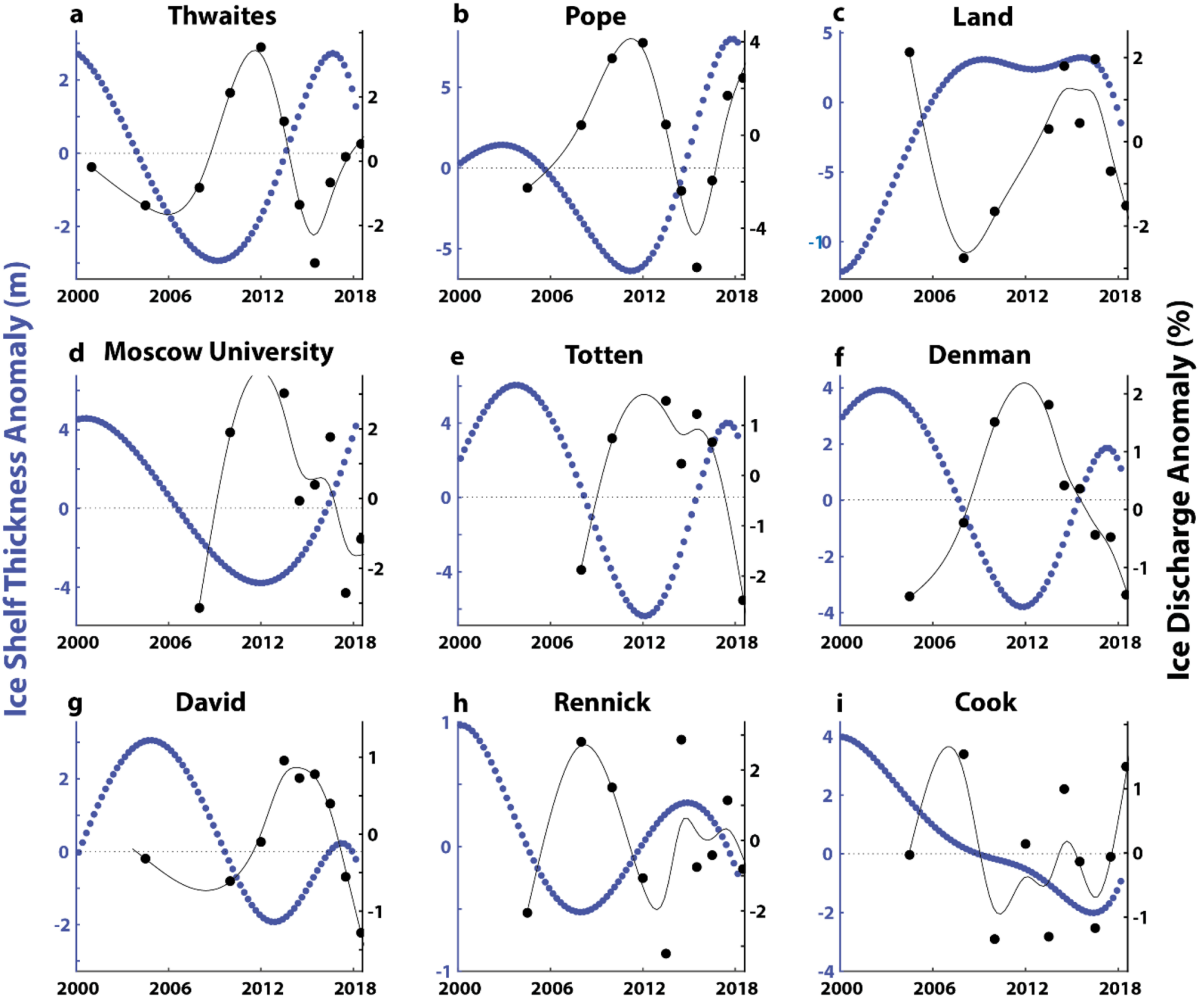
Linearly de-trended anomalies in ice-shelf thickness (m) versus linearly de-trended anomalies in ice discharge (%) for the (**a**) Thwaites, (**b**) Pope, (**c**) Land, (**d**) Moscow University, (**e**) Totten, (**f**) Denman, (**g**) David, (**h**) Rennick and (**i**) Cook East Glaciers. Note different scales on the *y*-axes and that the Cook catchment has been seperated between the East tributary and West tributray. This is because the West tributary does not have an ice shelf. The original ice-shelf thickness data has been taken from dataset produced in^16^.

We use a high-resolution ice-shelf thickness time-series^16^ to extract anomalies in ice shelf thickness and then compare these to ice discharge anomalies (see Methods). We focus on nine examples: the Thwaites and Pope Glaciers in the Amundsen Sea; Land Glacier in Marie Byrd Land; Moscow University, Totten and Denman Glaciers in Wilkes Land; and Rennick and Cook Glaciers in George V Land. We justify our selection of these example catchments because their ice shelves are large enough to be captured in the ice shelf thickness dataset^16^ and they represent a selection of the both warm-water ice shelves (Amundsen Sea, Marie Byrd Land and Wilkes Land) and cold-water ice shelves (George V Land). In general, we observe a clear pattern whereby periods of anomalous ice shelf thinning coincide with increases in ice discharge, while periods of anomalous ice shelf thickening coincide with decreases in ice discharge (Fig. 5). The only exceptions to this are at the Rennick (Fig. 5h) and Cook East Glaciers (Fig. 5i), where there is little relationship between ice shelf thickness anomalies and ice discharge. At Rennick, this might be explained by the comparatively small magnitude in ice shelf thickness variations (< 1 m). In contrast, at the Cook East Ice Shelf there are no clear inter-annual anomalies in ice shelf thickness, meaning any corresponding anomalies in ice discharge are not expected.

We interpret the periods of anomalous ice shelf thickening and negative ice discharge anomalies (e.g. in Fig. 5a–g) as a direct response to relatively cooler oceanic conditions and lower basal melt rates, whereas periods of anomalous thinning and increased ice discharge are linked to relatively warmer oceanic conditions and higher basal melt rates. At warm ice shelves, wind-driven variations in the transport of warm modified Circumpolar Deep Water (mCDW) onto the continental shelf have the potential to cause variations in basal melt rates underneath warm-water ice shelves^29–32^. These wind-driven variations in the transport of mCDW onto the continental shelf are linked to large-scale atmospheric patterns^33–35^ and, as a result, this mechanism can operate over a large spatial scale. This explains why multiple nearby neighbouring catchments can undergo similar patterns of interannual variability in ice discharge in the Amundsen Sea (Fig. 4d–g). The similar coherent response of outlet glaciers in Wilkes Land (Fig. 4r–t), where the continental shelf can also be flooded with mCDW is also indicative of a common large-scale atmospheric driver.

For cold-water ice shelves, inter-annual variations in basal melt rates are driven by high salinity shelf water (HSSW) and seasonal warming of the upper layers of the ocean near the ice front^16^. These variations in cold-water ice shelves can be linked to highly localised sea-ice and polynya processes^36–38^. These highly localised processes have the potential to drive a more localised response in ice discharge. For example, the variability in ice discharge at David Glacier is not observed in any of its neighbouring catchments. These processes have the potential to be driven by both external forcing e.g. katabatic winds^37,38^, but also internal ice sheet processes e.g. iceberg calving. For example, the calving of the Mertz Ice Tongue resulted in a large change in polynya persistency and resulting oceanic conditions^39,40^.

#### Ice shelf extent

**Figure 6:**
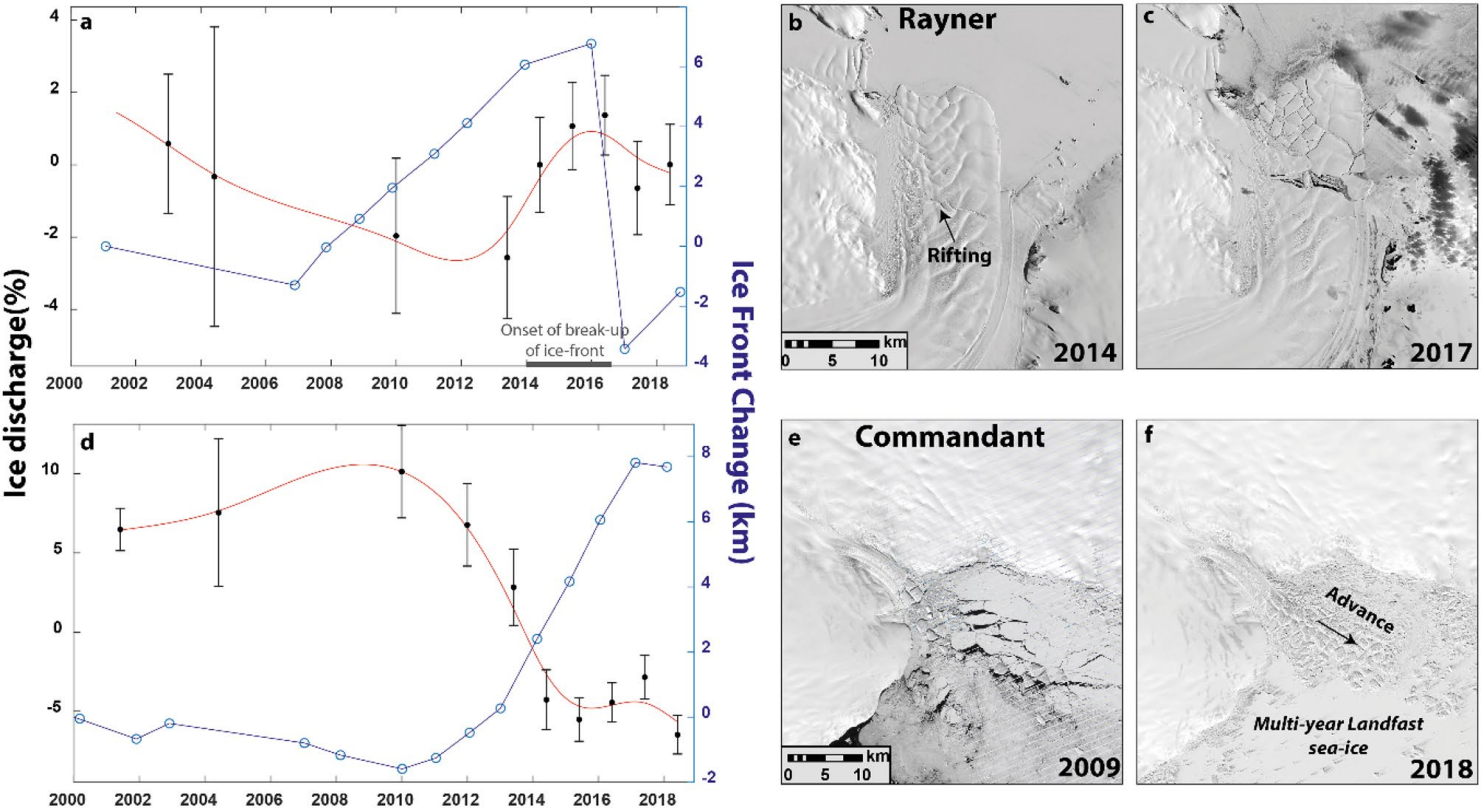
(**a**) Time-series of ice discharge change (%) and ice-front position change between 2000 and 2018 for Rayner Glacier. (**b**) and (**c**) are Landsat-8 images showing the progression of a calving event at Rayner between 2014 and 2017. (**d**) Time-Series of ice discharge and ice-front position change for Commandant Glacier, (**e**) and (**f**) are Landsat-7 (2009) and Landsat-8 (2018) images showing the rapid growth of the Commandant ice tongue in response to persistent landfast sea-ice. The red line in (**a**) and (**d**) are cubic spline trends of ice discharge.

Changes in ice shelf extent can directly influence ice discharge rates if dynamically important sections of floating ice are lost or enlarged^23^. An example of this process is seen at the Rayner catchment in Oates Land, where modelling experiments have shown that its entire floating ice shelf is dynamically important^23^ and where the observed inter-annual variation in ice discharge can be explained by its calving cycle (Fig. 6a). Between 2005 and 2014, the glacier advanced continuously while ice discharge decreased. However, between 2014 and 2016 ice discharge increased as the Rayner ice-front began to rift and break-up, before a final calving event in 2016 resulted in its ice front retreating ~ 10 km (Fig. 6a–c). After this event the ice front re-advanced and ice discharge started to decrease (Fig. 6a–c). It is important to note, however, that not all calving events result in an increase in ice discharge. If the calved portion is passive and offers limited buttressing, only a limited velocity response from ice inland would be expected^23,41^. For example, we detect no change in ice discharge rates following the calving of the Mertz ice tongue in 2010^42^.

Landfast sea-ice conditions can also play an important role in determining changes in ice shelf extent for some glaciers with heavily damaged ice shelves or ice tongues^43–46^. One of the most rapid changes in ice discharge was at the Commandant Glacier, a small glacier within the Adelie Coast catchment, where we observe a 15 ± 2% decrease between 2009/2010 and 2018 (Fig. 6d–f). This coincided with the formation of an ice tongue, of which growth continued unabated until the end of our observational period in 2018 (Fig. 6d). This is indicative of the growth of the ice tongue providing additional buttressing and driving a dynamic slow-down. The growth of the ice tongue appears to be anomalous. In all available satellite imagery since 1973, no comparable ice tongue is present, with the exception of a much smaller tongue in 1973 (Fig. S4). The anomalous ice tongue growth is, however, likely to be linked to an abrupt change in sea ice conditions. Prior to 2009 sea-ice cleared away each austral summer and sometimes resulted in small calving events, but post-2009 a band of multi-year landfast sea ice has formed which has remained consistently fastened to the ice-front and inhibited calving (Figs. S4, 6f). It is unclear what has triggered this abrupt change in sea-ice conditions, but we hypothesize a positive feedback whereby an initial cooler period enabled sea-ice to survive the summer and in doing so trapped detached icebergs in the embayment. These trapped icebergs may have then helped further strengthen the sea-ice by negating the impact of damaging oceanic swell^47–50^. Therefore, changes in ice shelf extent linked to changes in landfast sea-ice can provide a direct and rapid link between external forcing, ice shelf buttressing and ice discharge.

The link between landfast sea-ice and ice shelf extent may also be important in determining ice discharge variability for glaciers whose ice shelves are both damaged and float in confined embayments that are favourable for persistent landfast sea-ice formation. A 5 ± 2% reduction in ice discharge between 2011–2012 and 2015 at the western section of the Cook Glacier, for example, can be explained by a multi-year landfast sea-ice promoting ice-front advance^51^ and reducing ice discharge (Fig. S5). However, at the neighbouring Frost and Holmes catchments, which also underwent large calving events in response to landfast sea-ice break-up^44^, we do not observe any obvious relationship with ice discharge variability, indicating that the glacial ice lost was passive. We suggest that the interaction between landfast sea-ice and ice shelves may become a more important driver of variability in ice discharge in the future if ice shelves weaken and retreat into confined embayments and/or if landfast sea-ice were to decrease^52^.

#### Bed geometry

The response of a glacier to an initial velocity perturbation associated with a change in ice shelf buttressing is strongly modulated by the geometry of the glacier in its topographic setting. One potentially important aspect is the local slope of the topography at the grounding line. This is particularly the case for outlet glaciers with unconfined or weak ice shelves, which may be susceptible to rapid grounding line retreat along retrograde slopes^53,54^. However, for outlet glaciers with ice shelves that are able to provide sufficient buttressing, the local bed slope becomes less important in determining grounding line stability^27,55^. Therefore, if ice shelves are weakened sufficiently, local bed slope can be an important factor in determining grounding line migration. Retreat of the grounding line can cause further feedbacks because the associated loss in basal traction can lead to further acceleration and thinning^56,57^, thus creating a positive feedback. The acceleration associated with a loss in basal traction, in addition to reduction in ice shelf buttressing, has been shown to be an important factor in explaining the longer-term observed accelerations of both the Denman^58^ and Pine Island Glaciers^28^. Therefore, whilst changes in ice-shelf buttressing are likely to be the primary driver of the high spatial and temporal variability in ice discharge that we observe, the local bedrock slope at the grounding line is an important secondary factor in explaining the precise rate of response of individual glaciers.

**Figure 7:**
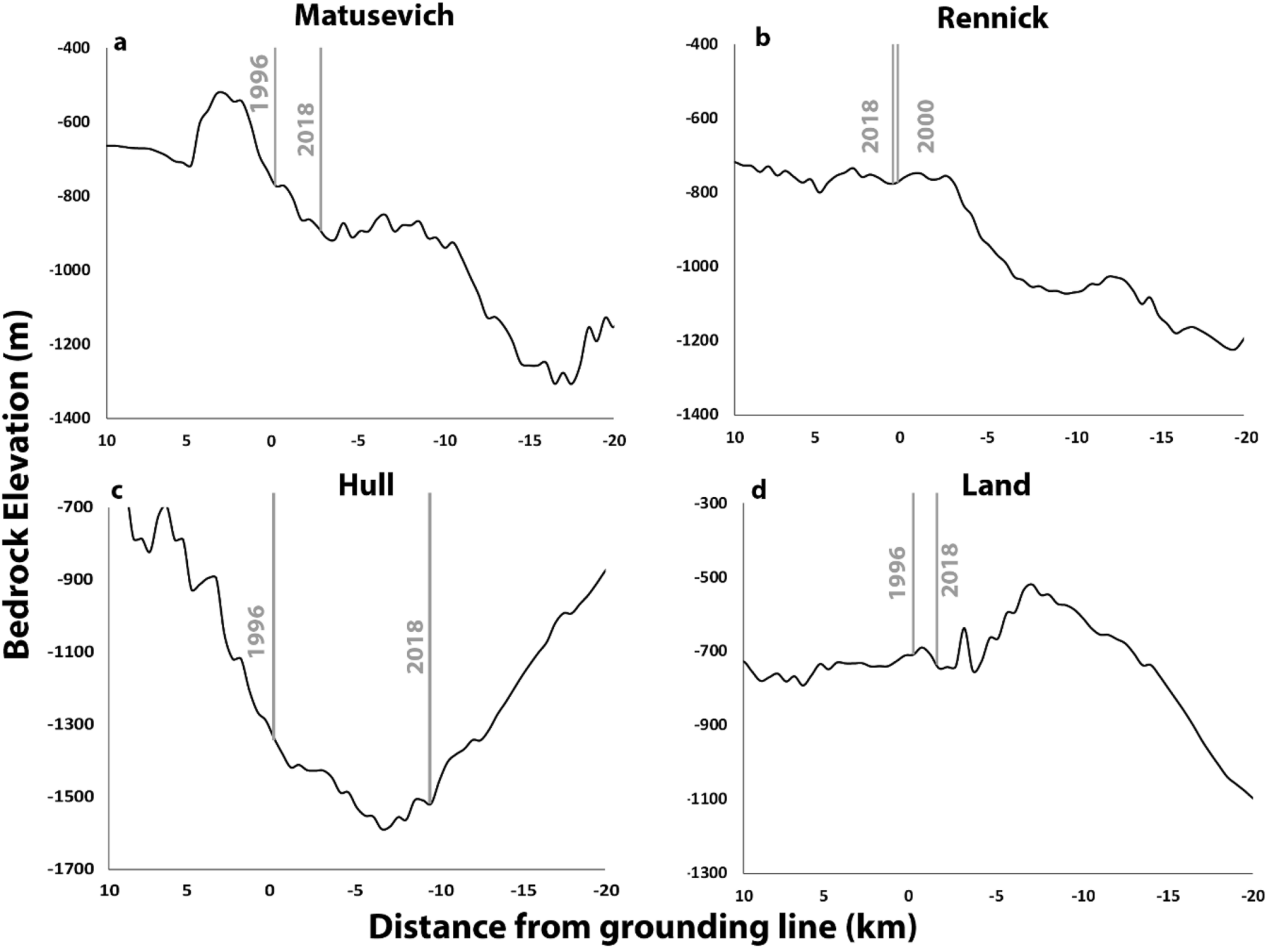
Bed topography profiles from BedMachine^14^ extracted along the central flow line of the (**a**) Matusevich, (**b**) Rennick, (**c**) Hull and (**d**) Land Glaciers. The grey vertical lines are InSAR derived grounding line positions^59,60^. On the x-axis, zero represents the earliest measured position of the grounding line, positive values are the bedrock elevations advanced of the grounding line and negative values are the bedrock elevation of grounded ice.

On a localised scale, variable bed topography may explain why some neighbouring catchments can simultaneously undergo opposing trends in ice discharge, when variations in external forcing might be expected to be similar. For example, the mostly consistent acceleration of the Matusevich (Fig. 4l) catchment between 2003/2006 and 2015 is anomalous amongst its neighbouring catchments (e.g. Cook, Slava, Rennick). A similar anomalous acceleration is also observed at the Hull catchment in Marie Byrd Land, where we observe a consistent increase in ice discharge throughout our observational period (Fig. 4h), but a much more varied discharge of the neighbouring Land Glacier (Fig. 4i). In both of these examples, the grounding lines of the glaciers displaying a spatiality anomalous acceleration, Matusevich (Fig. 7a) and Hull (Fig. 7c), have been retreating rapidly along retrograde slopes^59,60^. In contrast, there has been comparatively little grounding line migration in their respective neighbouring catchments, Rennick (Fig. 7b) and Land (Fig. 7d), which rest on a flat or prograde bedrock slopes. This would suggest that the spatially anomalous acceleration of Matusevich and Hull catchments has been strongly influenced by their underlying bed topography, which is conducive for rapid grounding line retreat.

## Conclusion

We have shown that much of the increase in AIS discharge between 1999 and 2018 took place in the first half of this period, with a 37 ± 11 Gt year^−1^ increase between 1999 and 2010 and a more limited change of only 4 ± 8 Gt year^−1^ between 2010 and 2018. This more limited increase in ice discharge since 2010 is linked to a more limited change in the Amundsen Sea sector and a decrease in ice discharge from Wilkes Land. Thus, the rate of acceleration in ice discharge of the AIS has not been consistent through time. At the scale of individual glaciers, we show widespread temporal and spatial variability across large sections of the ice sheet, sometimes resulting in neighbouring catchments undergoing opposing changes. Much of this variability is driven by changes in ice-shelf buttressing, which can be caused by changes in ice-shelf thickness and/or extent (e.g. major calving events). Variable ocean forcing can be linked to anomalies in ice shelf thickness, while changes in ice shelf extent can be linked to periodic calving cycles and landfast sea-ice induced changes. However, the specific response of outlet glaciers is further modulated by their unique geometries, which can amplify or dampen the response through various feedbacks. Overall, our results demonstrate a highly sensitive and complex pattern of ice sheet discharge that is evolving rapidly in response to external forcing and feedbacks associated with glacier geometry.

## Supplementary Information

Supplementary Information 1.

Supplementary Information 2.

Supplementary Information 3.

## Data Availability

The ITS_LIVE annual velocity mosaics are available at 10.5067/6II6VW8LLWJ7. The MEaSUREs annual velocity mosaics are available at 10.5067/9T4EPQXTJYW9. The MEaSUREs ice velocity mosaic of Antarctica (v2) is available at 10.5067/D7GK8F5J8M8R. The BedMachine dataset is available at 10.5067/C2GFER6PTOS4. The ice shelf thickness change dataset used in this study is available at 10.6075/J04Q7SHT. The MEaSUREs grounding line dataset is available at 10.5067/IKBWW4RYHF1Q and the Mohajerani grounding line dataset is available at 10.7280/D1VD6G. The Reference Elevation Mosaic of Antarctica (REMA) is available via the Polar Geospatial Center (https://www.pgc.umn.edu/data/rema/). MODIS imagery is available from the imagery from the NASA Worldview application (https://worldview.earthdata.nasa.gov). Landsat imagery was provided free of charge by the US Geological Survey Earth Resources Observation Science Center (https://earthexplorer.usgs.gov/).
