# Supplementary material for: High spatial and temporal variability in Antarctic ice discharge linked to ice shelf buttressing and bed geometry
Source: Sci Rep. 2022 Jun 29;12:10968. doi: 10.1038/s41598-022-13517-2 (PMC9243100; doi:10.1038/s41598-022-13517-2)
Supplement: Supplementary file 1 — Supplementary Information 1. [file 41598_2022_13517_MOESM1_ESM.pdf]

Supplementary Tables and Figures

Table S1: List of the ITS_LIVE and MEaSUREs annual velocity products used in each of the composite mosaics.

Epoch	ITS_LIVE	MEaSUREs
1999-2002	1999, 2000, 2001, 2002	
2003-2006	2003, 2004, 2005, 2006	
1999-2006	1999,2000, 2001,2002, 2003, 2004, 2005, 2006	2005-2006
2007-2008	2007, 2008	2006-2007, 2007-2008
2009-2010	2009, 2010	2008-2009, 2009-2010
2011-2012	2011, 2012	2010-2011, 2011-2012
2013	2013	2013-2014
2014	2014	2014-2015
2015	2015	2015-2015
2016	2016	2016-2017
2017	2017	
2018	2018	

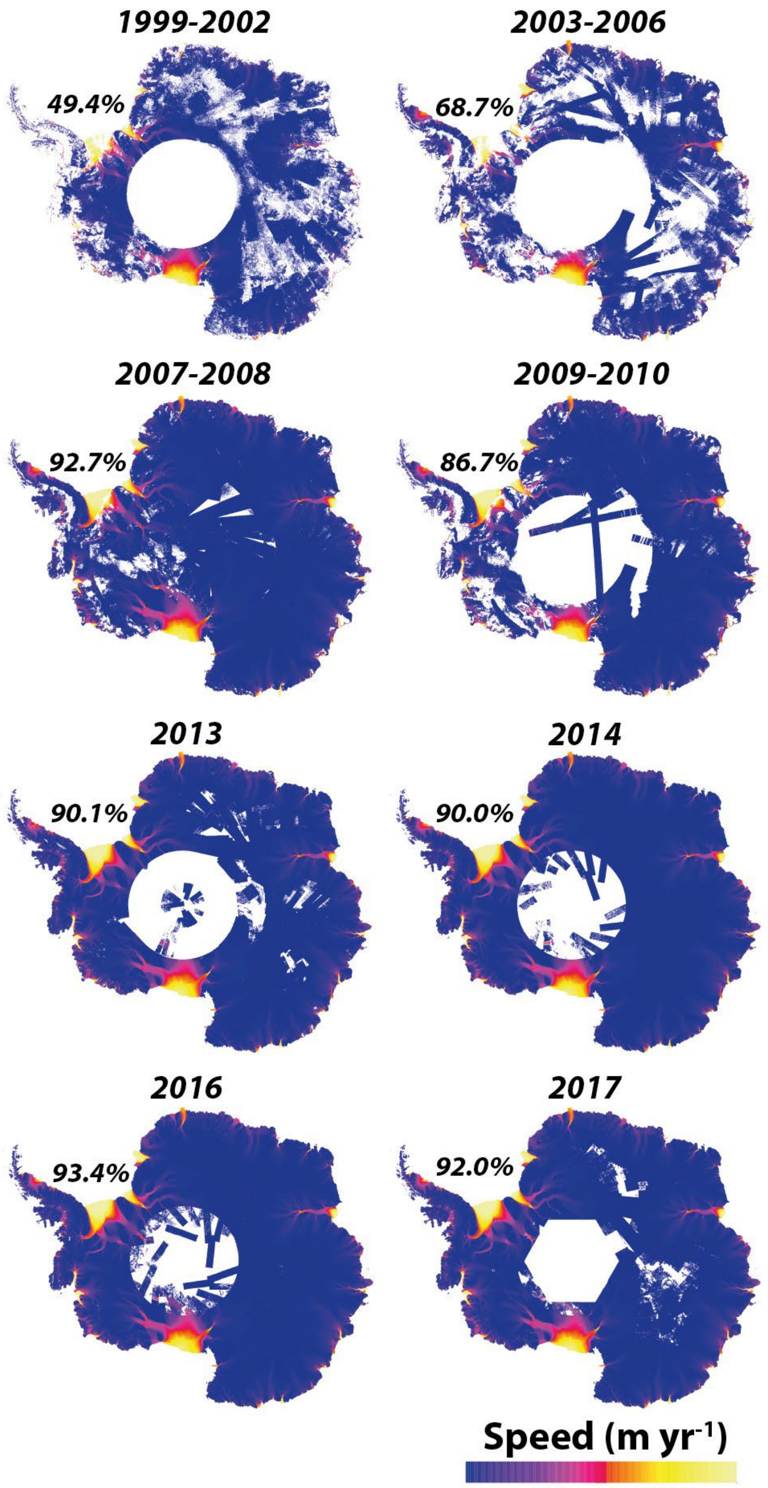

Figure S1: Spatial coverage of each mosaic composite used in our timeseries. The percentage on the top left of each mosaic represents the percentage coverage of ice discharge relevant to the MEaSUREs v2 (Rignot et al., 2011) ice velocity mosaic, which we used as a reference dataset.

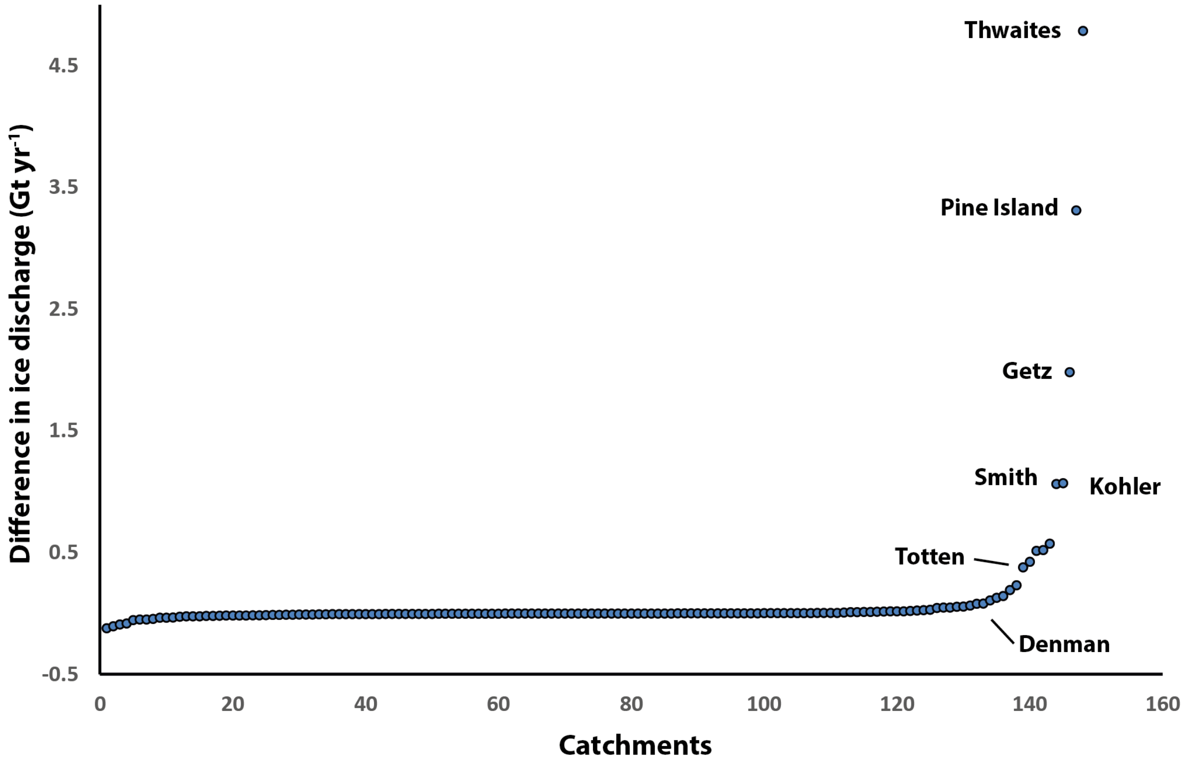

Figure S2: Difference in 2018 ice discharge between constant ice thickness and correcting ice thickness based on observed change between 2002 and 2018 for each glacier catchment. Catchments are ordered from minimum to maximum difference and selected catchments are labelled.

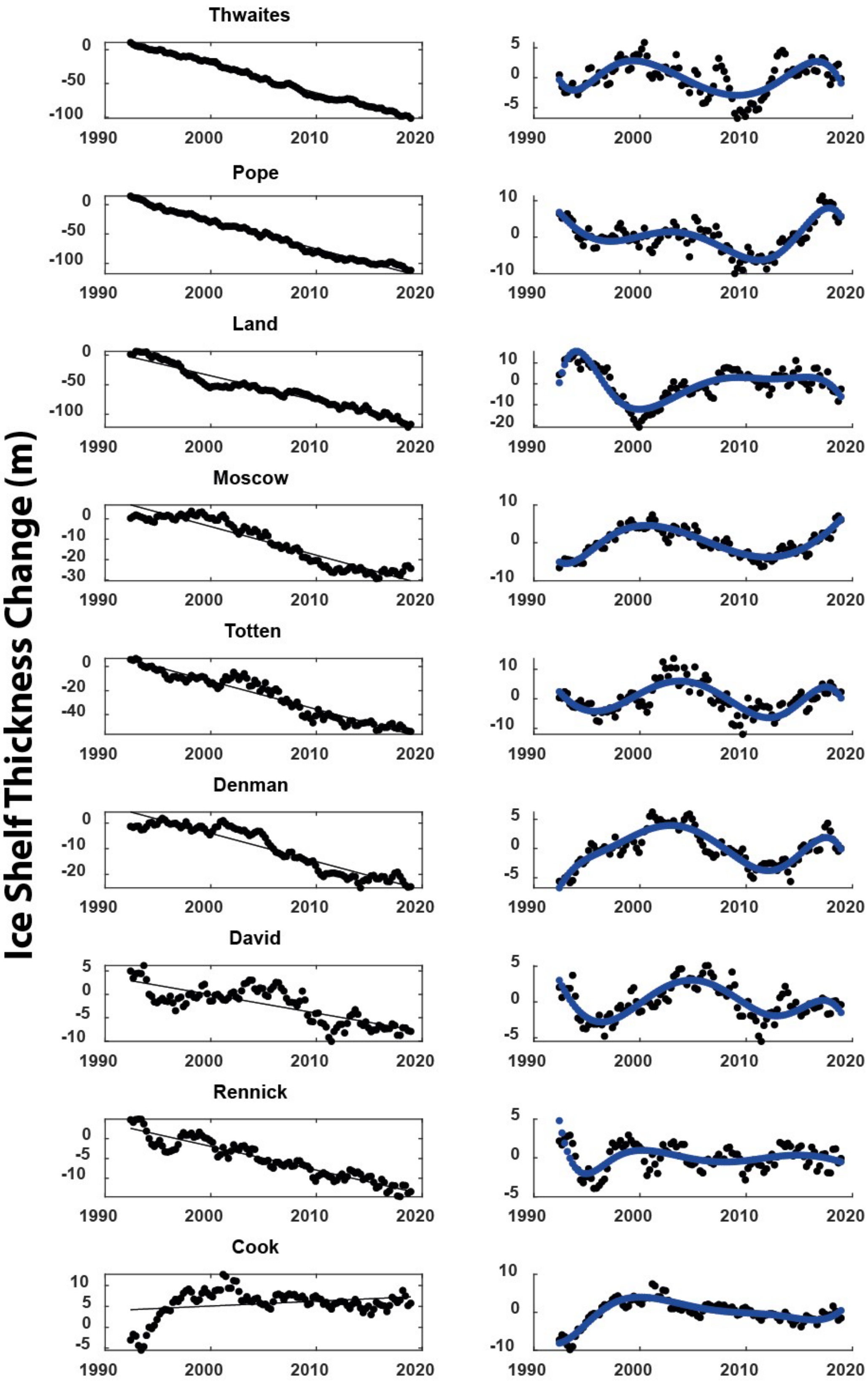

Figure S3: Ice shelf thickness change for the nine examples used in Figure 4. The left column represents the raw ice shelf thickness change data from Adusumilli et al. (2020). The column on the right is the linearly detrended anomalies (black dots) in ice shelf thickness change, fitted with a cubic spline (blue). The cubic spline is the data presented in Figure 4.

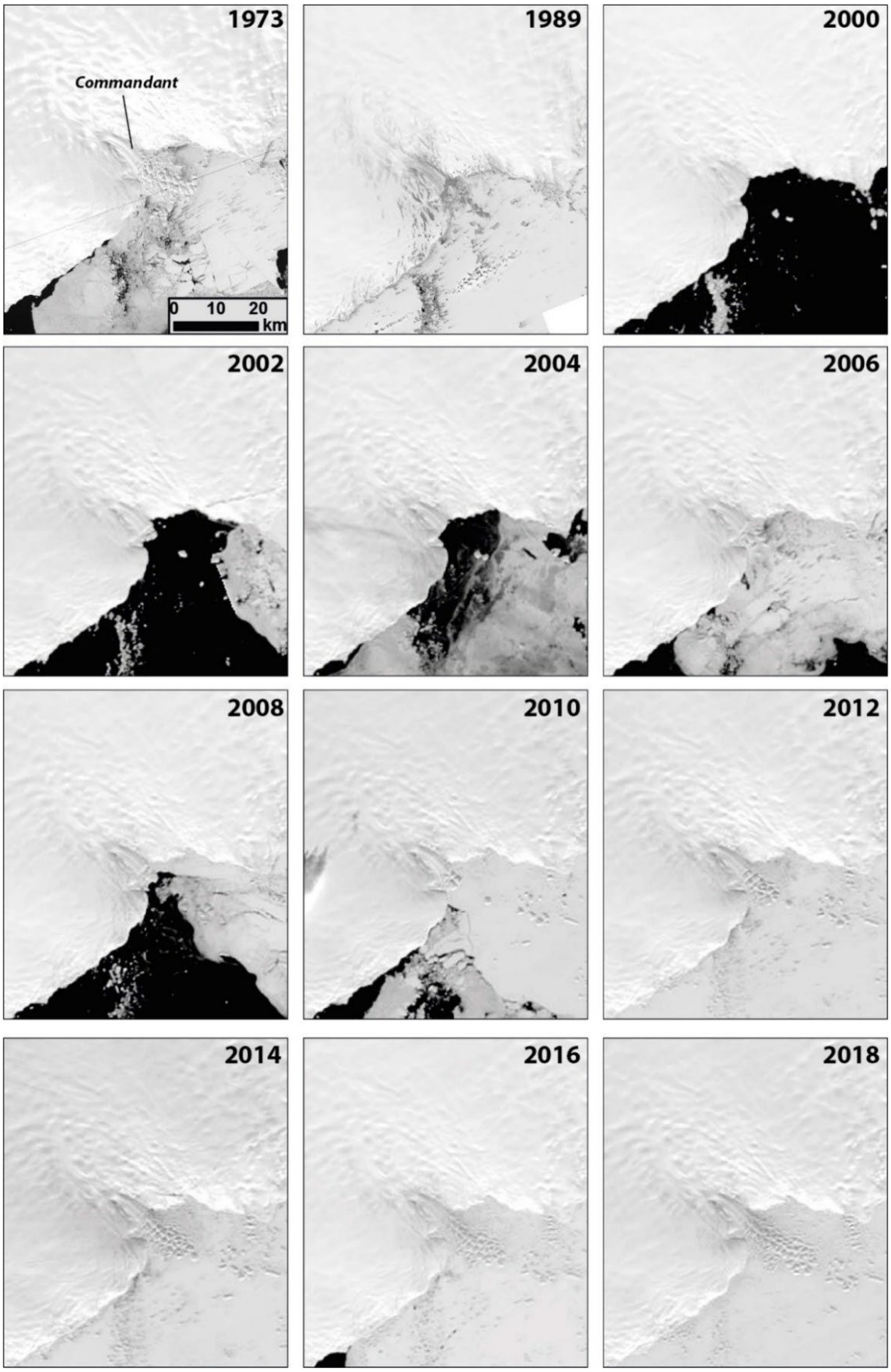

Figure S4: Satellite imagery of the Commandant Glacier. The 1973 and 1989 images are from Landsat-1 and Landsat-5 respectively. Images from 2000-2018 are MODIS images taken in March. The extent of the Commandant ice tongue in 2018 is the most extensive throughout the satellite record. Note from 2010 onwards the landfast sea-ice has remained present in each image.

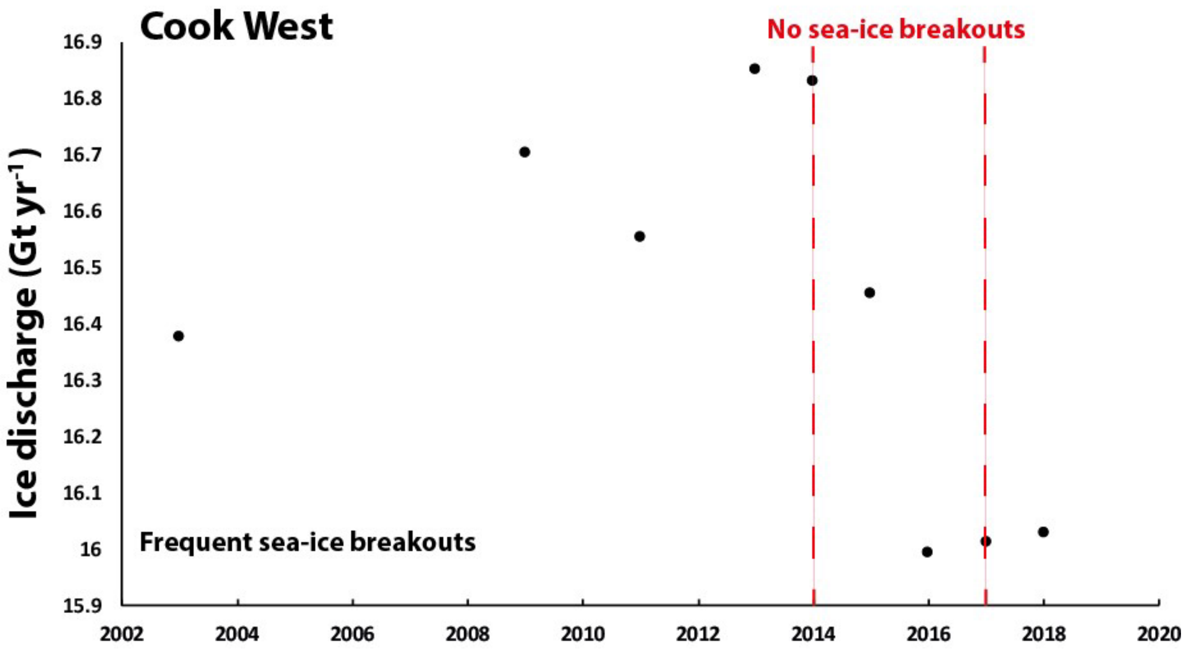

Figure S5: Timeseries of ice discharge from the Cook West Glacier. Note the decrease in ice discharge between 2014 and 2016 when landfast sea-ice remained fastened to the ice-front and prompted ice-front advance (see Miles et al., 2018).
